# Acellular Tissue Engineered Pericardial Patch Urethroplasty: A New Horizon of Substitution Urethroplasty

**DOI:** 10.5152/tud.2022.22018

**Published:** 2022-05-01

**Authors:** Sunirmal Choudhury, Eeshansh Khare, Dilip Kumar Pal

**Affiliations:** Department of Urology, Institute of Postgraduate Medical Education and Research (IPGME&R), Kolkata, India

**Keywords:** Urethral stricture, ATEPP, substitution urethroplasty, uroflowmetry

## Abstract

**Objective:**

Buccal mucosal graft is the best autologous material for substitution urethroplasty. However, in cases where buccal mucosa is unavailable, a non-autologous tissue like acellular tissue-engineered pericardial patch can be very helpful. Our study is a small approach regarding the success and durability of acellular tissue-engineered pericardial patch as a substitution tissue in urethroplasty.

**Material and methods:**

A total of 22 patients underwent acellular tissue-engineered pericardial patch substitution urethroplasty using dorsolateral onlay technique for long segment urethral stricture, for a period of two years. Observations and comparison were made in terms of postoperative change in maximum urinary flow rate (*Q*_max_), resolution of obstructive lower urinary tract symptoms, improvement in retrograde urethrogram and complications encountered, with buccal mucosal graft urethroplasty as a historical control.

**Results:**

Out of these 22 patients, 18 patients had successful outcomes considering maximum flow rate (*Q*_max_) > 10 mL/s on uroflowmetry, resolved obstructive lower urinary tract symptoms, and normal postoperative retrograde urethrogram, whereas four patients were considered a failure because of *Q*_max_ <10 mL/s, unresolved obstructed lower urinary tract symptoms and recurrence of urethral stricture on retrograde urethrogram and development of urethrocutaneous fistula.

**Conclusion:**

Acellular tissue-engineered pericardial patch substitution urethroplasty can be a useful alternative to autologous tissue substitution, especially where the buccal mucosal graft is unavailable for urethroplasty.

**Main Points**

- Postoperative *Q*_max_ improved in the majority of patients of acellular tissue-engineered pericardial patch (ATEPP) urethroplasty.
- Outcomes of ATEPP substitution urethroplasty were nearer to that of Buccal mucosal graft urethroplasty.
- Our results with ATEPP urethroplasty matched other studies using bovine pericardial patch for urethroplasty.

## Introduction

Urethral stricture is a well-known disease encountered by a urologist. There are various etiologies for urethral stricture and so their management. Medical treatment in the form of locally applied steroid^1^ and tacrolimus^2^ as well as surgical management in form of optical internal urethrotomy (OIU)^3^ and urethroplasty^4^ are available depending upon the length and nature of urethral stricture.

Urethroplasty is the mainstay of treatment for long segment urethral stricture. Urethroplasty may be anastomotic or substitution. Substitution urethroplasty uses flaps and grafts. Grafts used in urethroplasty can be autologous tissue like buccal mucosa, labial mucosa, lingual mucosa, and tunica vaginalis. Besides these, non-autologous tissue materials are also available in market. One of these is acellular tissue-engineered pericardial patch (ATEPP). In the present era of coronavirus disease (COVID) pandemic, harvesting autologous tissue from buccal mucosa may pose some risk to an operating urologist. This newer modality is now an interest of research in the field of urology and other medical fields, especially in cases where autologous tissue is not available for surgical management. A bovine pericardial graft for urethroplasty is integrative to native tissue,^5^ nonantigenic, cheap and readily available,^6^ flexible and durable,^7^ and easy to use. A tissue-engineered urethra can be constructed with a limited amount of material without harvesting a mass of autologous healthy tissue.^8^ In cases of long segment urethral stricture, buccal mucosal graft is most commonly used with a very good success rate. Harvesting a buccal mucosal graft causes great morbidity to the patient and also it is not available in all patients of urethral stricture, reasons may be the previous harvesting of this graft or unhealthy buccal mucosa (in cases of tobacco or betel nut chewers). In such cases, a non-autologous tissue graft can be a good substitute.

Very few studies are done for substitution urethroplasty using ATEPP. Here we want to share our experience of using a bovine pericardial patch in substitution urethroplasty in patients with long-segment urethral stricture.

## Material and Methods

This pilot study was conducted in a tertiary health care center in India for a period of two years after obtaining institutional ethical clearance (IPGME&R/IEC/2019/383). The aim of this study was to use ATEPP in patients undergoing substitution urethroplasty and find the outcome and also to compare the results with Buccal Mucosal Graft urethroplasty as a historical control. Inclusion criteria were all patients of long segment anterior urethral stricture (stricture length more than 3 cm involving penile and/or bulbar urethra), planned for substitution urethroplasty who are willing to give consent and do not meet the exclusion criteria. Exclusion criteria—patients with or without obstructive lower urinary tract symptoms (LUTS) with short segment strictures, having urinary tract infection, coagulation disorder, unable to lie in dorsal lithotomy position due to any musculoskeletal abnormality, local infection, bladder malignancy, and neuro-vesical dysfunction.

The total number of patients included in the study was 28. Out of these six patients were excluded from the final results as three patients lost follow-up and 3 patients withdrew during the study period. The final sample size was 22 patients. Patients included in the final sample size were evaluated in terms of relevant history (presence of obstructive LUTS), physical and local examination, and urological evaluation including uroflowmetry, ultrasonogram Kidney Ureter and Bladder (KUB) region with postvoid residual urine, retrograde urethrogram (RGU), and micturating cystourethrogram and urethroscopy. Out of these 22 patients, seven patients had histopathologically proven balanitis xerotica obliterans (BXO) changes. Two patients underwent elective suprapubic cystostomy (SPC) for resolution of upper tract changes prior to surgery. Two patients underwent SPC after surgery due to the development of urethrocutaneous fistula. After being diagnosed as a case of long segment urethral stricture as per aforementioned criteria, after proper counseling and getting written informed consent from all patients they underwent substitution urethroplasty using ATEPP in lithotomy position using midline perineal approach and dorsolateral onlay technique with unilateral urethral mobilization^9^ with preoperative sterile urine culture and pre-anesthetic fitness.

**Figure 1.**
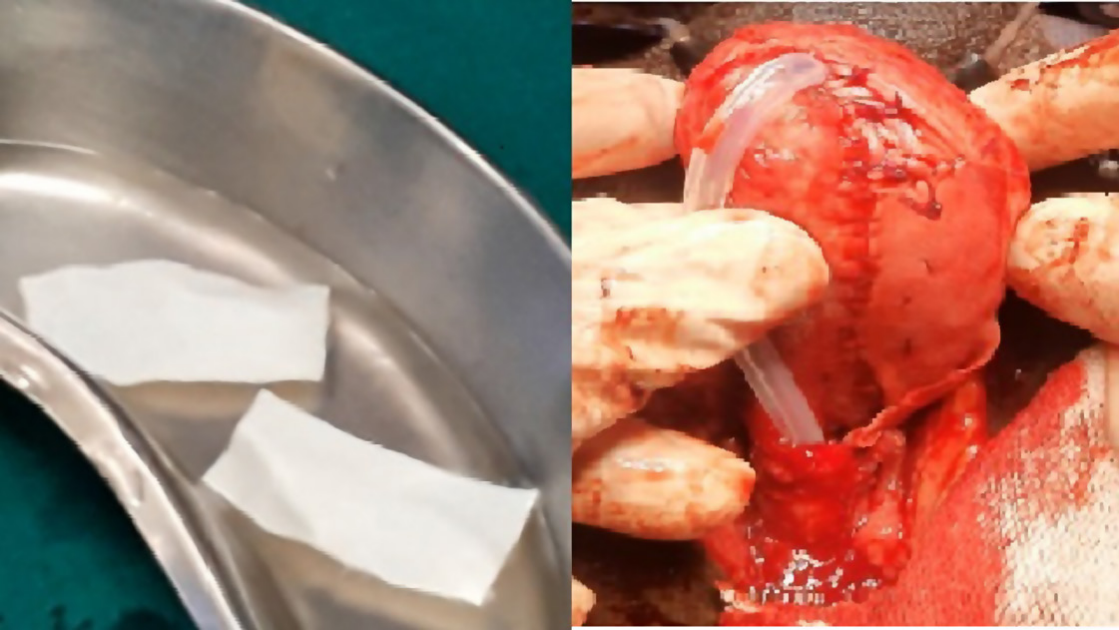
Acellular tissue engineered pericardial patch in normal saline (cut in two pieces) with quilted acellular tissue engineered pericardial patch with silicon Foley’s catheter in situ.

Acellular tissue-engineered pericardial patch (Figure 1) (brand name SYNCROSCAFF^®^ manufactured by SynkroMax Biotech Private Limited, India) available in sterile packing with dimensions of 4 × 4 cm and 6 × 6 cm with thickness 2-5 mm was used for substitution urethroplasty.

After dorsolateral mobilization^9^ of urethra on one side keeping the other side intact, the affected segment of urethra was opened dorsally. Appropriate-sized patches were cut according to the length of the stricture and the width of the native urethral plate with an intent to create the urethral lumen of 20Fr caliber. Acellular tissue-engineered pericardial patch was placed as a dorsal onlay patch. In stricture of length more than 5-7 cm, multiple patches (2 or 3 patches) were placed in continuous fashion along the urethral length. The patch was fixed and quilted with 4-0 vicryl sutures. Tubularization was done over a 16 Fr silicon Foley’s catheter. The perineal wound was closed in layers after placement of a suction drain which was removed on the second post-operative day. Perioperative and post-operative antibiotics and analgesics were given up to the seventh post-operative day.

Success was determined on the basis of postoperative maximum urinary flow rate (*Q*_max_) >10 mL/s, resolution of obstructive LUTS, and post-operative normal RGU.

### Statistical Analysis

For statistical analysis, data were entered into a Microsoft Excel spreadsheet and then analyzed by SPSS version 27.0 (IBM SPSS Corp.; Armonk, NY, USA) and GraphPad Prism version 5. A chi-squared test (χ^2^ test) was any statistical hypothesis test wherein the sampling distribution of the test statistic was a chi-squared distribution when the null hypothesis was true. Without other qualifications, the “chi-squared test” often was used as short for Pearson's chi-squared test. Unpaired proportions were compared by chi-square test or Fischer’s exact test, as appropriate. Z-test (Standard Normal Deviate) was used to test the significant difference in proportions. *P*-value ≤ .05 was considered as statistically significant.

### Observation and Result

**Figure 2.**
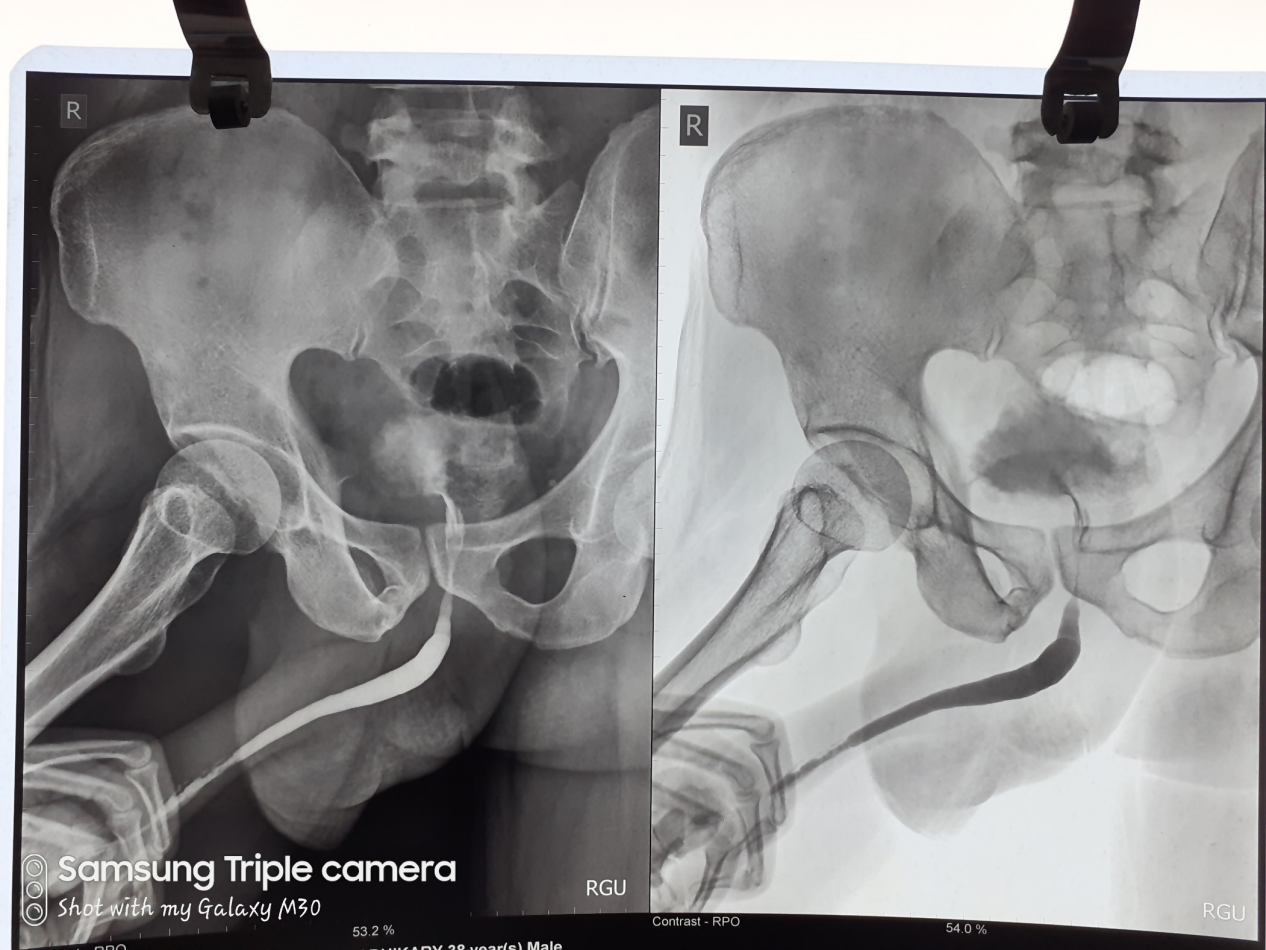
Preoperative retrograde urethrogram showing penile urethral stricture.

**Figure 3.**
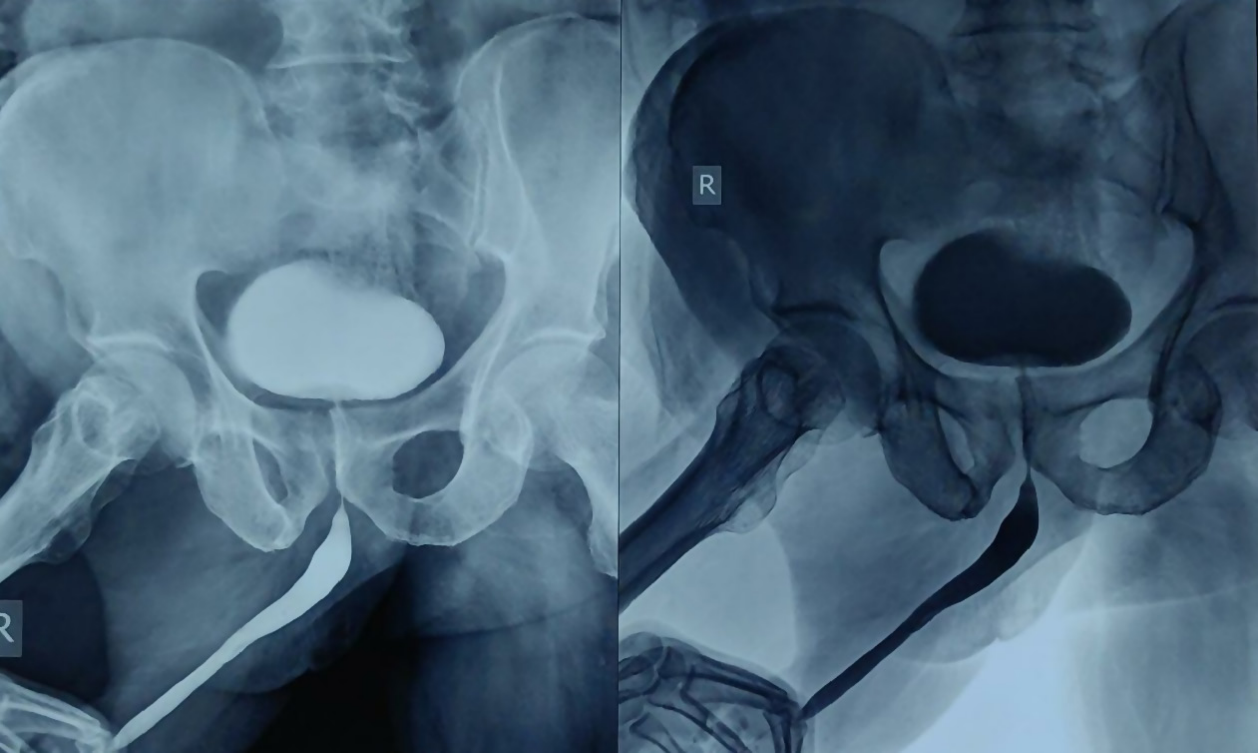
Postoperative retrograde urethrogram showing normal urethral calibre.

A total of 22 patients underwent ATEPP substitution urethroplasty for long segment urethral strictures after being properly counseled about the surgical procedure. The patient’s age varied from 24 years to 66 years with mean and median age of 41.5 years and 45 years, respectively. Fourteen patients out of 22 patients were tobacco or betel nut chewers and had poor oral hygiene and unhealthy buccal mucosa. Six patients were recovered patients of COVID. Seven patients had histologically proven BXO changes. Two patients had a history of buccal mucosal graft urethroplasty 5 years back. One patient had a history of anastomotic urethroplasty two years back. The location of the stricture on the basis of RGU and urethroscopy in this study was as follows: Penobulbar in 12 cases and long segment penile urethral stricture in seven cases, mid with distal bulbar urethral stricture in three patients (Table 1). The average stricture length was 10 cm ranging from 5 cm to 17 cm (Table 1). The width of the native urethral plate at the site of stricture ranged from 4 to 8 mm with an average of 6 mm. Preoperative maximal flow (*Q*_max_) on uroflowmetry was between 3.6 mL/s and 10 mL/s (Table 2) with a mean and median value of 6.34 mL/s and 6.8 mL/s, respectively. All patients underwent ATEPP substitution urethroplasty (Figure 2) via perineal approach using dorsolateral onlay technique. The average duration of surgery was 3.29 hours. Catheterization was done with silicone 16 Fr Foley’s catheter (Figure 3) in all patients. The average duration of catheterization was 3.86 weeks, after which the catheter was removed in the outpatient department (OPD) and uroflowmetry was done. Later on, patients were followed up on an outpatient department (OPD) basis at three months and six months post-operatively. Post-operative maximum flow rate (*Q*_max_) on uroflowmetry ranged from 7.2 mL/s to 25.6 mL/s with a mean and median of 18.26 mL/s and 16.4 mL/s, respectively, at 6 months. Post-operatively RGU was done in all patients with relapse or persistence of obstructive symptoms and urethroscopy was done only in patients and in *Q*_max_ <10 mL/s.

Out of 22 patients, 18 (81.82%) were considered as a success and 4 (18.20%) were classified as a failure on the basis of recurrence of stricture on RGU and decrease in uroflowmetry and persistence or recurrence of obstructive LUTS. *Q*_max_ > 10 mL/s on uroflowmetry at 6 months was considered as a success measure in our study. A positive correlation was found between the size of the urethral plate and pre-operative *Q*_max_ but the result was not statistically significant (*P*-value .238) (Table 3). A positive correlation was found between the size of the urethral plate and post-operative *Q*_max_ at per urethral catheter removal (Table 3). Similarly, a positive correlation was found between the size of the urethral plate and post-operative *Q*_max_ at 3 months and 6 months but the result was significant with an improvement in *Q*_max_ at both these postoperative duration (Table 3). A negative correlation was found between length of stricture and pre-operative *Q*_max_ and the result was statistically significant (*P*-value .003) (Table 3). Also, a negative correlation was found between length of stricture and post-operative *Q*_max_ after urethral catheter removal, at 3 months and at 6 months, however, the results were not statistically significant in terms of decrease in *Q*_max_ (Table 3).

Out of 18 patients, three patients developed short narrowing in the bulbar urethra (<1 cm) at 3 months on RGU for which OIU was done for 1 patient, and per urethral catheter was kept for 1 week and removed thereafter. Two patients had passable narrowing in the penile urethra for which endodilatation was done and catheterization was done for 1 week and removed thereafter. On further follow-up at 6 months, these three patients had satisfactory uroflow rates (*Q*_max_ >10 mL/s) and were considered successful. Failure was defined as a *Q*_max_ of < 10 mL/s on uroflowmetry. Four patients had *Q*_max_ of <10 mL/s, at 6 months follow up, and the lowest *Q*_max_ of which was 7.2 mL/s at 6 months. Out of these four patients with *Q*_max_ <10 mL/s, two patients who had a past history of buccal mucosal graft urethroplasty developed urethrocutaneous fistula at 3 months follow up which on urethroscopy came out to be having complete urethral obliteration at the distal penile urethra. The other two patients had histologically proven lichen sclerosus where one patient had wound infection leading to graft necrosis in the post-operative period, initially managed with wound dressing and antibiotics and underwent lay open urethroplasty and another patient had a poor flow of urine postoperatively at six months which on further evaluation on RGU and urethroscopy was found to have panurethral narrowing and was considered a failure.

## Discussion

The buccal mucosa is routinely used for substitution urethroplasty^10^ but many times it is found to be unhealthy, especially in those who chew tobacco and betel nut which cause submucosal fibrosis and hyperkeratosis and thus rendering oral mucosa unsuitable for harvesting. Harvesting of buccal mucosa also causes morbidity in the patients. There are also complications associated with buccal mucosal harvesting like pain, numbness, difficulty in opening the mouth, chewing, and eating. As a urologist operating on COVID recovered patients with substitution urethroplasty using buccal mucosal graft may pose some risk to the urologist during harvesting. So in such cases, there is a need to find a tissue material for substitution urethroplasty and ATEPP can be the one needed.

There are efforts going on in different parts of the world for use of new tissue or patch for urethroplasty as a replacement for buccal mucosa. Porcine small intestine submucosa (SIS) is also a readily available acellular matrix used in substitution urethroplasty for urethral stricture in many human studies with long-term safety and efficacy.^11,12^ These matrices provide structural support to cells and have the ability to induce the ingrowth of urothelium, smooth muscle, nerve cells, and endothelium and thus providing a method of neovascularization after graft placement, promoting enhanced survival of the graft. The increased cost in xenograft material production, along with the risk of disease transmission and variable mechanical strength, are some of its limitations for clinical use. The decellularization and sterilization techniques can denature proteins in the extracellular matrix, thus damaging the physiological conditions for graft uptake. A tissue-engineered bovine pericardial graft in comparison with decellularized porcine SIS matrix is integrative to native tissue, nonantigenic, cheap and readily available, flexible and more durable, and easy to use. The delayed risk of calcification and glutaraldehyde-mediated decreased recellularization are some of its limitations.

Our study with acellular pericardial patch is a new horizon in this direction. The mechanism of ATEPP is that it acts as a scaffold for the regeneration of urothelium in the urethra, while completely getting absorbed into the native tissue. It was found that ATEPP, an acellular material comprising of pure collagen, may provide a natural micro-environment for host cell migration and proliferation, and tissue regeneration.^13^ There are few research work with tissue-engineered patch mostly in animal model.^14,15^

There is a case report of successful repair of an enterovesical fistula by use of bovine pericardium.^16^ Bovine pericardium is widely used in surgery and cardiovascular surgery. Bovine pericardial patches have several advantages compared to prosthetic patches, like superior biocompatibility, easy handling, less suture line bleeding, and reduced infection rates.

BMG urethroplasty is considered a standard procedure by many urologists for anterior urethral stricture as it has a very good success rate. But in cases where buccal mucosa is unavailable, the tissue-engineered bovine pericardial patch can provide similar outcomes as in our study with a success rate of 82%.

In a study by Lara et al^17^ found that the bovine pericardium may be an alternative option in the treatment of urethral lesions in dogs however with increased urethrocutaneous fistula formation (80%) but the rate of urethrocutaneous fistula formation was much lesser in our study (9%). According to Bhargava et al^18^, despite initial complications like fibrosis and contracture, tissue-engineered graft may be a useful autologous material which can replace urethral tissue. In our study two patients developed complete urethral obliteration, one patient had graft necrosis within three months post-surgery and was considered a failure. Another study by Mandal T et al^19^ reported promising results of substitution urethroplasty using a bovine pericardial patch with a success rate of 89%, however, the success rate in ours was 82%. The average median pre-operative and post-operative *Q*_max_ in the study by Mandal et al^19^ were 5 mL/s and 24 mL/s, respectively. Likewise in our study average mean pre-operative and post-operative *Q*_max_ was 6.8 mL/s and 16.4 mL/s, respectively. In our study, 4 patients were considered as surgical failure in comparison to one patient in a study by Mandal et al^19^ (Table 4). In comparison to buccal mucosal graft urethroplasty, substitution urethroplasty with ATEPP also shows good results. In a study by Sami Mahjoub Taha Awad et al^20^ (Table 4), their success rate with buccal mucosal graft urethroplasty was 90% with a 10% re-stricture rate. In our study success rate with ATEPP urethroplasty was 82% with an 18% failure rate. Another study by Spilotros et al^21^ (Table 4) showed a success rate of 81% and 19% failure rate with buccal mucosal graft urethroplasty.

In our study, we used ATEPP as an alternative to oral mucosal graft in these 6 COVID-19 recovered patients, and all of them had successful results.

The short duration, small sample size, and limited follow-up of the patients were few limitations of our study.

In clonclusion, we hereby conclude that ATEPP substitution urethroplasty as a promising alternative to buccal mucosal graft urethroplasty with its tissue-engineered properties and can be considered as a new horizon to substitution urethroplasty.

## Figures and Tables

**Table 1.** Success and Failure Rate According to Stricture Site, Stricture Length, and Urethral Plate Size

**Patients **	**Number (%)**	**Success (%)**	**Failure (%)**
Total	22(100)	18(81.82)	4(18.20)
**Site of stricture **			
Penobulbar	12(54.54)	10(45.45)	2(9.09)
Long segment penile	7(31.82)	5(22.72)	2(9.09)
Midbulbar + distal bulbar	3(13.64)	3(13.64)	0
			
**Length of Stricture (in cm)**			
5-7	6(27.27)	5(22.73)	1(4.54)
8-11	6(27.27)	5(22.73)	1(4.54)
12-14	7(31.82)	6(27.27)	1(4.54)
>14	3(13.64)	2(9.09)	1(4.54)
			
**Size of urethral plate (in mm)**			
4	2(9.09)	1(4.54)	1(4.54)
5	10(45.45)	8(36.36)	2(9.09)
6	7(31.82)	6(27.27)	1(4.54)
8	3(13.64)	3(13.64)	0

**Table 2.** Change in *Q*_max_ After Per Urethral Catheter Removal, 3 Months and 6 Months in Relation to Length of Stricture and Size of Urethral Plate

**S no.**	**Size of Urethral Plate (mm)**	**Length of Stricture (cm)**	**Site of Stricture**	**Pre-Operative ** *Q* **max** ** (mL/s)**	**Post-operative ** *Q* **max** ** (mL/s)**		
**At Per Urethral Catheter Removal **	**3 Month**	**6 Months**
1	5	12	Penobulbar	6.4	19.6	18.4	18.5
2	5	10	Penobulbar	5.2	21.7	20.4	20
3	6	12	Penobulbar	5.4	23.2	24.6	21.5
4	5	7	Penobulbar	10	17.6	14.6	8.6
5	4	14	Long penile	7.6	15.2	13	7.4
6	5	15	Long penile	4.4	21	22	21
7	8	5	Mid and distal bulbar	6.8	22.8	23.8	24.5
8	6	8	Penobulbar	8.4	23	24.8	25.6
9	5	14	Long penile	6.2	18.5	15.4	15.6
10	8	6	Mid and distal bulbar	6.4	22.8	24.6	23.8
11	6	7	Penobulbar	6	22.6	20.4	21.1
12	5	12	Long penile	7.2	24.5	22.6	21.5
13	6	8	Penobulbar	8.5	22.5	23.4	21.5
14	5	12	Penobulbar	6.8	26.6	19.6	20
15	6	7	Long penile	5.6	22.4	23.8	23.4
16	5	14	Penobulbar	4.8	21.5	23	22
17	5	17	Penobulbar	3.6	22.6	24	23.5
18	4	15	Long penile	3.8	14.6	14	7.2
19	5	10	Long penile	5.6	23.4	13.8	8.2
20	6	11	Penobulbar	6.4	25.6	22.5	21.6
21	6	10	Penobulbar	6.6	23.8	24.5	23.2
22	8	5	Mid and distal bulbar	7.8	25.6	26.3	22.6

**Table 3.** Correlation of Size of Urethral Plate and Length of Stricture with *Q*_max_

**Parameters **	**Correlation Coefficient/** *P* ** with Size of Urethral Plate**	**Remarks**	**Correlation Coefficient/** *P* ** with Length of Stricture (cm)**	**Remarks**
Pre-operative *Q*_max_ (mL/s)	Pearson correlation coefficient (*r*): 0.262	Positive correlation	Pearson correlation coefficient (*r*): 0.595	Negative correlation
*P* = .238	Not significant	*P* = .003	Significant
Post-operative *Q*_max_ after per urethral catheter removal	Pearson correlation coefficient (*r*): 0.550	Positive correlation	Pearson correlation coefficient (*r*): −0.358	Negative correlation
*P* = .008	Not significant	*P* = .102	Not significant
Post-operative *Q*_max_ at 3 months	Pearson correlation coefficient (*r*): 0.689	Positive correlation	Pearson correlation coefficient (*r*) −0.340	Negative correlation
*P* < .0001	Significant	*P* = .122	Not significant
Post-operative *Q*_max_ at 6 months	Pearson correlation coefficient (*r*): 0.624	Positive correlation	Pearson correlation coefficient (*r*) −0.289	Negative correlation
*P* = .002	Significant	*P* = .191	Not significant

**Table 4.** Comparison of Success and Failure Rate Between ATEPP and Other Studies

**Study**	**Type of Tissue Used**	**Success (%)**	**Failure (%)**
Mandal et al^19^	Bovine pericardial patch	89	11
Sami Mahjoub Taha Awad et al^20^	Buccal mucosal graft	90	10
Spilotros et al^21^	Buccal mucosal graft	81	19
Our study	ATEPP	82	18

**ATEPP, **acellular tissue-engineered pericardial patch.
